# Towards a comprehensive structural coverage of completed genomes: a structural genomics viewpoint

**DOI:** 10.1186/1471-2105-8-86

**Published:** 2007-03-09

**Authors:** Russell L Marsden, Tony A Lewis, Christine A Orengo

**Affiliations:** 1 Department of Biochemistry and Molecular Biology, University College London, Gower Street, London WC1E 6BT, UK

## Abstract

**Background:**

Structural genomics initiatives were established with the aim of solving protein structures on a large-scale. For many initiatives, such as the Protein Structure Initiative (PSI), the primary aim of target selection is focussed towards structurally characterising protein families which, so far, lack a structural representative. It is therefore of considerable interest to gain insights into the number and distribution of these families, and what efforts may be required to achieve a comprehensive structural coverage across all protein families.

**Results:**

In this analysis we have derived a comprehensive domain annotation of the genomes using CATH, Pfam-A and Newfam domain families. We consider what proportions of structurally uncharacterised families are accessible to high-throughput structural genomics pipelines, specifically those targeting families containing multiple prokaryotic orthologues. In measuring the domain coverage of the genomes, we show the benefits of selecting targets from both structurally uncharacterised domain families, whilst in addition, pursuing additional targets from large structurally characterised protein superfamilies.

**Conclusion:**

This work suggests that such a combined approach to target selection is essential if structural genomics is to achieve a comprehensive structural coverage of the genomes, leading to greater insights into structure and the mechanisms that underlie protein evolution.

## Background

In order to move towards a complete understanding of the biochemical functions and their mechanisms of action within the cell, structural biology faces the task of characterizing the shapes and modes of action of the entire protein repertoire encoded within the genomes. However, with the rapid growth in the number of known genome sequences and the relatively tiny number of experimentally solved protein structures, it is of considerable importance to develop efficient strategies to structurally and functionally annotate sequence space.

The combined advances in the late 1990s of methods such as X-ray crystallography, Nuclear Magnetic Resonance (NMR), gene cloning and expression, and whole genome sequencing, signalled the advent of structural genomics, enabling the goal of high-throughput protein structure determination to become a feasible proposition. As a result a considerable number of structural genomics projects have been initiated around the world [1,2] and though each may differ in their absolute objectives, all work upon the principle of achieving high-throughput structural determination with a view to gaining novel insights into protein function from a structural perspective. Structural genomics has now become a driving force behind new developments in protein structure prediction technology, aiming to automate, and consequently expedite, all areas of the experimental pipeline, ultimately benefiting the structural biology community as a whole. Recent analyses of structures released by the initiatives have highlighted the significant contribution they are now making in both the scope and depth of our structural knowledge of protein families, especially when compared to the relative contribution of non-structural genomics structures. The worldwide structural genomics initiatives now contribute approximately half of new structurally characterised families and over five times as many novel folds as mainstream structural biology, despite accounting for only ~ 20% of the new structures [3,4].

Through the use of homology modelling methods to extend the value of each newly solved structure across 'sequence space' (a term used here to describe all known protein sequences), it is not unreasonable to expect structural genomics to make far reaching advances into the structural landscape over the coming years. Recent advances in sensitive sequence comparison algorithms, homology modelling and threading methods [5-8] mean that it is not necessary to experimentally characterise the structure of every protein – a procedure clearly limited in terms of time and cost. Evolutionary related proteins share similar structures [9], and in cases where one or more members of a related set of sequences, or domain family, has been structurally characterised, structural data can be transferred to the remaining structurally uncharacterised family members. The accurate one-to-many structural annotation of protein sequences is fundamental to gaining a significant structural coverage across the genomes.

Amongst the structural genomics projects that were instigated to work upon this principle is the Protein Structure Initiative (PSI), funded by the National Institute for General Medical Sciences in the United States [10,11], which aims to target new areas of structure space for which an experimental structure had not yet been solved. In so doing, it is hoped that each structure will maximally cover surrounding sequence space by acting as a structural template for comparative modelling and fold recognition [12-18]. The PSI project has recently moved into its production phase where it is hoped that approximately 3000 new structures will be solved in its five year duration [19].

Structural genomics target selection, the procedure through which specific proteins are selected for structural characterisation, often views sequence space in terms of its organisation into protein domain families. That is, collections of evolutionarily related sequences that can be prioritised according to a range of properties, such as size, taxonomic distribution and suitability of family representatives for high-throughput structure determination [20-23]. Sequence comparison methods, such as Position Specific Scoring Matrices or hidden Markov models, are now sensitive enough to group distantly related sequences into a *'coarse-grained' *classification of domain families. For example, domain-level family annotations of the genomes can be achieved using hidden Markov model libraries derived from domain structure classifications such as the SCOP [24] and CATH [25] databases and domain sequence classifications such as Pfam [26].

Through the use of such coarse-grained family annotations one can examine sequence and structure space at the domain-level in order to quantify the number and types of, as yet, structurally uncharacterised domain families. For instance, from a structural genomics viewpoint it is of considerable interest to calculate the number of experimental structures that will be required to structurally annotate all protein sequences. Clearly such findings can vary depending on how domain families are constructed (i.e. the number of families (or granularity) is dependent on the quality of homology-models required) and how sequence-structure coverage is calculated (e.g. as a percentage of sequences or percentage of residues that have a near or distant structure homologue) [27-32].

Such estimates generally focus on coarse-grained coverage where domain sequences have been grouped through the identification of distant relationships. Domain families for which a structural representative has yet to be experimentally solved (i.e. a structurally uncharacterised family) can be identified and prioritised according to the number of family-members, where the benefit of solving a structure for a given family can therefore be seen to correlate with family-size – the greater the family size, the greater the structural coverage. For example, recently Chandonia and Brenner [30] proposed the Pfam5000 target selection strategy which suggested that target selection could be guided through the selection of a manageable number of target proteins from a list of the largest 5000 Pfam families, many of which lack a member of known structure.

Targeting sequence space through coarse-grained target selection provides an important directed approach to characterising structural representatives of protein families. However, other analyses, including our own [32], have also conceded that structural-coverage based on a coarse-grained measure will be limited in terms of reliable structural and functional annotation. The fraction of proteins in a given genome for which we can infer structures by homology modelling depends on the accuracy required for a model. For instance, high-accuracy models require high levels of sequence identity between target and template sequences, with lower measures of similarity simply providing the basic description of protein fold. The level of granularity required for the selection of additional targets should therefore account for the number of sequences that can be computationally modelled with 'useful' accuracy from each solved structure. It is generally accepted that sequences sharing 30% or more sequence identity are likely to share a similar fold [9] and accordingly, to confidently construct models of reasonable accuracy, at least 30% sequence identity (60% overlap) must be shared between the sequence to be modelled and the structural template [33]. Reliable functional inference is even more limited with 60% or more sequence identity required [34-36]. The selection of *'fine-grain' *targets from within larger coarse-grained families of distantly related proteins would provide a more thorough coverage of functional space as it relates to protein structure [37].

Furthermore, one must also consider the number of structurally uncharacterised families that are within the scope of the experimental methodology used by structural genomics. The principle aim of structural genomics initiatives is the high-throughput determination of protein structure. Achieving such levels of structure solution has required the development of automated protein structure determination pipelines [38,39]. However, predicting protein behaviour within these pipelines is still problematic especially considering the fact that high-throughput methods require well-expressing and highly soluble proteins. Accordingly, the cloning and expression of target proteins is often parallelized in order to increase the chances of producing sufficient protein that is not only soluble but ultimately amenable to X-ray crystallography or NMR structure analysis [40-43]. Strategies include the cloning of target sequence homologues from as wide a range of sequenced organisms as possible, often described as a multi-orthologue approach. Historically, most structural genomics targets tend to be prokaryotic in origin allowing direct amplification from genomic DNA. Large-scale expression of eukaryotic proteins is much more challenging and considerably more expensive and therefore has not become routine in structural genomics, although several centres are developing new methodology [19].

In this work we consider how a broad structural coverage of the genomes might be achieved and the limitations that may be encountered in a family-based target selection procedure. A principle aim of Phase 2 of the PSI (PSI2) structural genomics initiative is to solve structures for coarse-grained families which do not yet have a representative of known structure. We aim to identify the number of these families and what level of additional structural coverage will be achieved if structural genomics devotes much of its efforts towards solving the largest (in terms of sequence members) of these families. We also ask what proportion of these coarse-grained families are within reach of structural genomics pipelines – specifically those employing a multi-orthologue approach focused on solving prokaryotic sequences. We also consider what benefits might be achieved if structural genomics forfeited the opportunity to solve 'novel-folds' with a view to solving additional structures for large structurally-diverse families. We ask how this would compare to pursuing structurally characterised coarse-grained families in terms of structural coverage and species distribution.

Through a comprehensive analysis of CATH, Pfam and NewFam [32] domain families we find that many of the largest structurally uncharacterised domain families are eukaryotic or viral and have no prokaryotic sequence members. Therefore these families may well be more challenging targets. In addition, many of the coarse-grained families which do have prokaryotic relatives have relatively few members, offering small returns from a structural annotation viewpoint.

We show that solving structures for many of the largest fine-grained subfamilies (derived through sequence clustering at 30% sequence identity) from structurally characterised families can offer similar increases in sequence coverage, with more available prokaryotic sequences for potential high-throughput structure determination. Such an approach could be used in concert with the targeting of structurally uncharacterised families to achieve a broad coverage of sequence space. For instance one could identify those cases where a structurally characterised family member exists (e.g CATH, SCOP families), but reliable modelling coverage of the remaining family is low, particularly in the case of many large families of protein domains which can display considerable divergence in their molecular function [34,37,44].

We evaluate the current structural coverage of domain families in Swiss-Prot and TrEMBL sequence databases [45], which encompass a wide representation of known sequences, using the CATH domain structure classification. An additional mapping of the manually curated Pfam-A and our in-house automatically-derived NewFam domain family supplement [32] (described in Methods), is then made in order to comprehensively identify the number and distribution of remaining structurally uncharacterised domains and corresponding families. Structural coverage can be calculated by a number of criteria; we consider the coverage of these families across Swiss-Prot and TrEMBL using three measures, as a percentage of sequences, domains and residues.

From these observations we show that whilst targeting structurally uncharacterised domain families may achieve small gains in structural coverage compared to existing structural coverage, such efforts will expand our knowledge of protein folds and function. Furthermore, PSI2 structural genomics has the potential to solve structures for around half the remaining structurally uncharacterised families accessible to multi-orthologue approaches. We also demonstrate that identifying additional targets within fine-grained subfamilies from broad, structurally characterised families, often with a wide species distribution, will enable comparable increase in structural and functional coverage, whilst expanding our knowledge of these highly expanded protein families. The proportion of effort that should be spent on solving structurally uncharacterised families or re-sampling from large structurally characterised superfamilies should be addressed as the initiatives progress.

## Results and discussion

### Domain family annotation of protein sequences

In order to measure what contribution structural genomics must make in order to provide broad structural annotation coverage of protein families we have based our calculations on the CATH, Pfam and NewFam protein domain-level annotations (as outlined in the Methods). Accordingly, our first aim was to assign a comprehensive coverage of protein domains to the sequences held in the Swiss-Prot and TrEMBL sequence databases. By organising sequence data into domain families we are able to quantify those families lacking structural coverage, or large families with limited structural representation. In addition, remaining unassigned sequence regions to which no family assignments can be made must also be accounted for, as they still represent a significant proportion of the genomes. Definitions of the datasets and terms used in this analysis can be seen in Table 1.

**Table 1:** Definition of terms.

**Term**	**Definition**
**SP-trEMBL**	Sequence dataset containing 2,241,227 sequences from the Swiss-Prot (version 48.1) and TrEBML (version 31.1) sequence databases.
**Integr8_263**	Sequence dataset containing 913,094 sequences from 263 completed genomes listed in the Integr8 genome database.
**Pfam_struc**	Pfam-A family containing a PDB structure that has not yet been classified into the CATH domain database.
**NewFam**	Protein families generated in the Gene3D

The classification of proteins into families is most commonly carried out at the domain level (e.g. SCOP, CATH, Pfam, SMART [46]) since it is well recognised that the protein domain represents the evolutionary building blocks from which larger multi-domain proteins have been constructed via domain duplication and recombination events. From a structural perspective, domains can be viewed as independent units of protein folding, whilst from a sequence perspective they tend to be considered as recurring units of evolution. Despite the difference in definitions, in many cases the domain families generated by each system are equivalent [47]. Those domains belonging to the same family share a common protein structure and, depending on their degree of relatedness (i.e. how the family has evolved), can share similarities in their function.

Structural domain assignments were made using HMMs [48] based on the CATH domain database (version 3.0). Of the 2,241,277 non-redundant sequences (greater than 50 residues in length) in SP-TrEMBL (Swiss-Prot version 48.1, TrEMBL version 31.1), 1,101,819 sequences (49.2%) were assigned to one or more CATH domain families. Whilst this figure may be considered to represent a rough guide to the extent to which the known fraction of whole protein sequences are covered in whole or by part by structure annotation, it does not account for 1. Domains within these sequences that cannot be structurally annotated, 2. Protein Data Bank (PDB) [49] structures not yet classified in the CATH domain database and 3. The quality of structural models that can be obtained (i.e. only represents a coarse grained coverage).

To extend the domain coverage beyond the CATH domain assignments we assigned Pfam-A domain families (version 19) bringing the total number of sequences in SP-TrEMBL with one or more domain family assignment to 75.0 % (1,681,640 sequences). Finally, assignments using NewFam families, available from the Gene3D database (see Methods and [32]), were used to further extend domain family assignments across the remaining unannotated sequence regions. As such, 79.1% of sequences in SP-TrEMBL sequence database could be assigned to one or more domain families containing 2 or more sequences.

### Removal of families unsuitable for structure determination

When calculating existing and additional structural annotation coverage of domain sequences that might be achieved by structural genomics, it is important to identify those sequences that are unlikely to be tractable to high-throughput structural characterisation. It is generally agreed that in order to reduce the high attrition rate encountered in high-throughput structural genomics pipeline, sequences with low complexity regions, coiled-coils and helical transmembrane helices are best avoided. Such features can be reliably predicted using computational methods, and sequences or families with a significant proportion of 'problematic' residues (see Methods) can be removed from the target list. In Table 2 we show the breakdown of these categories across the SP-TrEMBL sequences, 268 completed genomes annotated by the integr8 database [50] and the model genome examples. Over 18% of the domain sequences in SP-TrEMBL are considered problematic and excluded from high throughout structural characterisation. Just 13% of domains in the compact genome of *T. maritima *appear as problematic for structure determination, though another prokaryotic genome, *B anthracis *appears to have the highest level of potentially intractable domain sequences (nearly 20%). It is worth noting that such prediction methods only offer a rough measure for the exclusion of problematic sequences. Parameters that accurately linked sequence composition and features to the bottlenecks in structure characterisation, such as protein expression, solubility and crystallisation, would be of considerable value to structural genomics target selection. It is also of importance to note that a domain-based approach to high-throughput structural characterisation brings its own difficulties in terms of resolving domain boundaries that enable the expression of soluble protein.

**Table 2:** Percentage of problematic and singleton domain sequences.

	**Percentage of domains**	
**Sequence dataset**	**Transmembrane & problematic**	**Singleton**

SP-trEMBL	18.5	22.6
integr8_263	17.9	24.9
*A thaliana*	17.5	16.0
*B anthracis*	20.3	8.6
*C elegans*	19.8	22.1
*D melanogaster*	18.7	18.7
*E coli*	15.7	7.3
*H sapiens*	15.9	20.9
*S cerevisiae*	14.9	24.7
*T maritima*	13.4	12.7

The percentage of problematic and singleton domain sequences in Swiss-Prot & TrEMBL, 263 completed genomes and eight model genomes Problematic domains are defined as those containing helical transmembrane helices or significant regions of low complexity or coiled-coil

Table 2 also shows the percentage of 'singleton' domain sequences within SP-TrEMBL and model genomes. The structural characterisation of true singleton sequences would offer large insights into the uniqueness of each species, providing a more comprehensive understanding of the structure-sequence relationship where they can be assigned as very remote homologues to known structural families. However, by definition, their structural characterisation would also provide a small modelling leverage across the genomes. Additionally, their species-specific nature reduces the chance of achieving a successful structural characterisation since they cannot be characterised through a multi-orthologue approach. The percentage of singleton sequences varies considerably between genomes (e.g. 7% in *E. coli *compared to over 22% in the eukaryote *C. elegans*). By our definition, the proportion of singleton sequences calculated in this analysis is subject to the proportion of domain sequences that are assigned to CATH, Pfam-A and NewFam domain families, and therefore is partially dependent on the sensitivity of assignment of these domain families using HMM methods. The true nature of singleton or 'ORFan' sequences has been open to much debate [31,32,51,52]. It has been suggested that their existence is partly due to the sparse sampling of sequence space (and that over time, sequence relatives will be found), or that many of these sequences relate to miss-predicted non-expressing proteins. It still appears somewhat unclear as to whether the number of singleton sequences will rise, or fall as more genomes are completed and their gene maps revised. Additionally, in this study the percentage of singleton sequences is related to the length threshold used to include unassigned regions. We cannot be certain that all unassigned regions are indeed true protein domains (or multi-domains), however in mind of the fact that structural genomics target lists tend to avoid small fragment sequences we apply a cautious length threshold of 80 residues (compared to 50 residues) for inclusion of unassigned regions into our domain-level coverage calculations.

### Protein sequence coverage by current structural data

Our attention now turns to the proportion of sequences to which some structural data can already be assigned. We identified 2486 coarse-grained CATH and Pfam_struc domain families already containing one or more PDB structures (see Methods). Table 3 summarises the coverage statistics across all sequences in the SP-trEMBL dataset and also the 263 completed genomes using three measures of coverage: First, on the basis of per-sequence coverage, calculated as the fraction of *whole-protein *sequences with at least one domain belonging to a given structurally characterised family. Secondly, per-domain coverage, where the fraction of all *domain *sequences belonging to a given structurally characterised family is calculated. Thirdly, coverage is calculated in terms of the fraction of *residues *assigned to a structurally characterised family (where all residues between the N and C-termini of an HMM alignment are included).

**Table 3:** Current structural coverage of sequences, domains and residues in Swiss-Prot & TrEMBL

**Coverage type**	**Percentage coverage**					
	
	**Per sequence**		**Per domain**		**Per residue**	
	Integr8_263	**SP-trEMBL**	Integr8_263	**SP-trEMBL**	Integr8_263	**SP-trEMBL**

**All Sequences**	52.4	**54.4**	44.8	**47.7**	34.5	**36.2**
**- excluding transmembrane & problematic sequences**	/	**/**	53.4	**57.3**	41.1	**44.1**
**- excluding transmembrane problematic & singleton sequences**	/	**/**	71.1	**81**	59.5	**63.8**

For all measures a slightly lower percentage structural coverage is found across the 263 completed genomes (integr8_263 dataset) compared to SP-TrEMBL. Of the three measures, per-sequence calculations give the highest levels of coverage, with 54.4% of SP-TrEMBL sequences containing one or more domains (52.4% of completed genomes) that can be assigned to a coarse-grained family that is already structurally characterised. This is an overestimate of our current ability to structurally annotate the genomes, because it does not account for the fact that many protein sequences (up to 80% in eukaryotes) contain two or more domains, and as yet, many of these domains sequences cannot be classified into a structurally characterised family (upon which we base our structural coverage calculations). Accordingly we have attempted to calculate structural coverage on a per-domain basis. Whilst one cannot always accurately predict the domain content of a given sequence (robust domain boundary prediction is an as yet unsolved challenge) we have, where necessary, estimated the number of protein domains within a given sequence/genome (see Methods). In so doing, we calculate that 47.7% of domain-like sequences in SP-trEMBL are structurally annotated at a coarse-grained level, a lower but possibly more realistic view. Calculating coverage on a per-residue basis shows that 36% of residues in SP-TrEMBL fall into the 2486 CATH and Pfam_struc families identified in this analysis.

In these values we account for every sequence, predicted-domain and residue in our sequence database. However, the high-throughput nature of structural genomics generally requires that sequence or families with a significant percentage of transmembrane regions or 'problematic' regions, such as low-complexity or coiled-coils should be excluded from the target selection process. In Table 3, we also show coarse-grained structural coverage expressed as a percentage of domains and residues that are expected to be tractable to structural genomics, i.e. transmembrane and problematic domains are excluded from this calculation. These values suggest that we currently have at least fold-level annotations for 57.3 % of domains and 44.1% of residues that are tractable to high-throughput methods. Even so, it is important to note that the remaining 42.7% of domains may still not be tractable to structural genomics because, for example, we do not consider components of complexes, low-expression, poor-solubility proteins etc.

Finally, in view of the suggestion that structural genomics should aim to solve structural representatives of sequence families, we show coverage excluding transmembrane, problematic sequences and also excluding singletons. Such a calculation strips-out a large proportion of sequence space, focusing coverage on areas accessible to structural genomics, with over 80% of 'accessible' non-singleton domain sequence already having some coarse-grained structural coverage (63.8% of residues). However, such values are misleading if one considers the goal of achieving a comprehensive structural and functional annotations of the genomes. Accordingly, we use per-domain coverage of *all *predicted domain sequences for the remaining calculations in this study.

### Additional structural coverage

In order for structural genomics efforts to provide increased levels of structural coverage it is logical that target lists should favour representatives from the largest coarse-grained sequence families for which no structure has yet been acquired. Indeed, such approaches to target selection have been discussed in various analyses [27-31], including the Pfam5000 (Chandonia and Brenner) which suggested that structural genomics should aim to solve structures such that each of the 5000 largest non-membrane protein Pfam-A families (and therefore of significant biological interest) includes one or more structural representative. With almost over half of the 5000 largest Pfam-A already having a structural representatives, this would require approximately 2500 additional structures to achieve this goal (a structure count that is considered to be within the capability of the production phase of the Protein Structure Initiative) [17].

**Figure 1:**
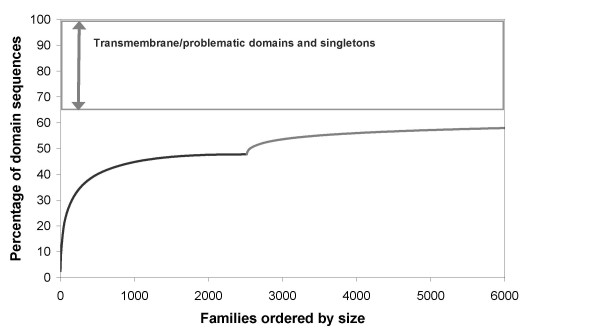
Coarse-grained structural coverage of domain sequences in Swiss-Prot-TrEMBL Families are ranked in order of size, largest to smallest The black line represents coverage of domain sequences by 2486 CATH and Pfam-A_struc families, whilst the grey line represents additional coverage that would be achieved by solving a structure for structurally uncharacterised Pfam-A and NewFam domain families.

In Figure 1 we show the coarse-grained sequence coverage of domain sequences in structurally characterised families, followed by the coverage of structurally uncharacterised domain families. Domain families are ordered according to their size (the number of domain sequence relatives identified by HMM searches), largest to smallest and coverage is given as the percentage of all domain sequences, including problematic sequences and singletons. We identify 2486 CATH and Pfam-A_struc families, covering just over 47% of domain sequences. Assignment of Pfam-A and NewFam domain families, over and above the CATH family annotations, identified 4100 structurally uncharacterised Pfam-A and over 50,000 structurally uncharacterised NewFam families. Solving a new structure for a comparable number of these largest non-structural families (the vast majority of which are Pfam-A families) would increase coverage of SP-TrEMBL by 10%, a relatively small fraction compared to current coverage levels, revealing that we already have structural representatives for many of the largest domain families.

### Historical structural coverage of Pfam

**Figure 2:**
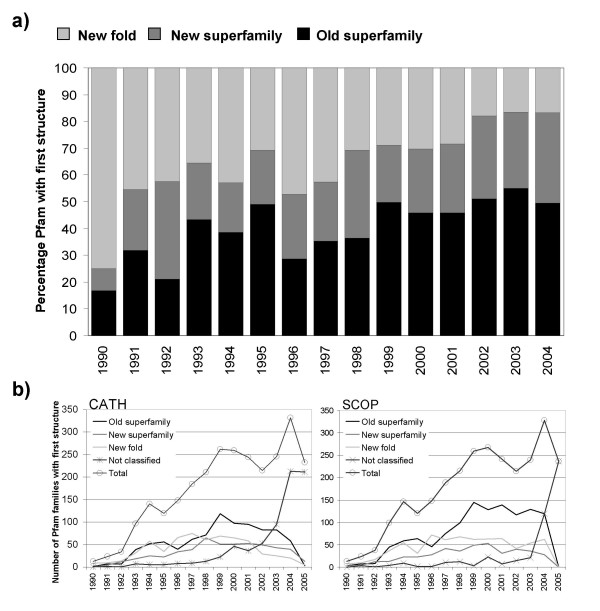
**a) **The percentage of newly solved Pfam-A families, per year, classified as a new fold, new superfamily, or old superfamily in the CATH domain classification Values are calculated as the percentage of first-solved structures that have been classified in the CATH database **b) **The underlying data used in this calculation The calculated number of new folds and superfamilies are similar when using either the CATH or SCOP domain classifications.

Using the CATH domain structure classification, we considered the extent to which newly structurally characterised Pfam-A domain families tend to represent a novel CATH fold or a founding member of a CATH superfamily using the historical trend observed in the Pfam database. In Figure 2a we show the percentage frequency for which the first structure solved for a given Pfam-A family represented a previously unobserved fold or an old fold, but the first member of a CATH domain superfamily. Values are given as the percentage of these first-solved structures that are classified in the CATH database. On average, since 1990 172 Pfam families have gained their first structure each year (average of 255 per year since 2000). Between the years 1990 and 2005, the fraction of first-solved structures that are novel folds (as classified by CATH) has gradually reduced (from 75% to 17%) though the number of newly structurally characterised families has increased from 13 in 1990 to an average of 332 in 2004. From these figures it appears that an average 50% of newly structurally characterised Pfam-A families in the last 5 years can be seen to correspond to an experiment on protein domain belonging to a new superfamily if not a novel fold.

Figure 2b shows the source data in more detail for structures classified in the SCOP and CATH domain databases respectively. It can be seen that both databases fall behind in their classification of first-solved Pfam structures in the latter years. Despite this fact, when calculating values as a percentage of *all *first-solved structures, on average, approximately one third of the domains represent a new fold or new superfamily in SCOP in the early part of this decade (e.g. 1999–2003). It will be of considerable interest to repeat this analysis on the next releases of the CATH and SCOP databases. These data suggest that the pursuit of structures for uncharacterised sequence families, such as Pfam, is likely to yield structural characterisations that represent significant and interesting variations of known folds and also a fair percentage of novel folds.

### Structural coverage of model genomes

**Figure 3:**
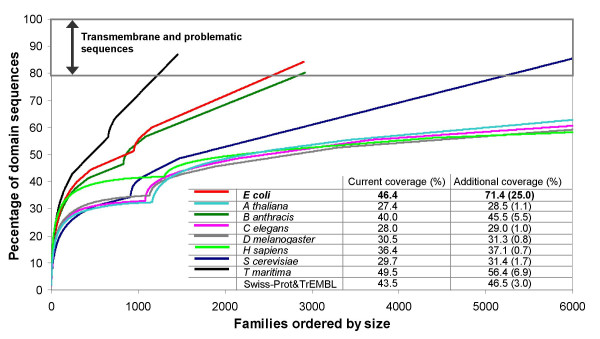
Coarse-grained structural coverage of seven model genomes. The benefit of solving a structure for 1571 structurally uncharacterised families in *E. coli *across the remaining six genomes is shown in the inset table.

The current coarse-grained structural coverage of domain families in eight completed 'model' genomes, followed by the coverage of non-structural Pfam-A and NewFam families is shown in Figure 3. Indeed a single-genome approach to structural genomics appears attractive because of the possibility of identifying the minimal component of genes necessary for life. For many of the genomes illustrated, solving structures for 2000 additional families (within reach of PSI2) would result in almost a doubling of structural coverage. However, a single-genome approach to structural genomics has its drawbacks. Many of the sequences in a given genome tend to belong to small families with little or no overlap with other genomes, whilst in addition, up to 20% of sequences may be classed as singletons (i.e. specific only to a given species). By our calculations *E. coli *contains 1571 domain families with no solved structure. Characterising a structure for each of these families would increase structural coverage in *E. coli *from 42% to 70% however the leverage of these new structures upon the other model genomes is comparatively small, Figure 3 inset table.

### A closer look at structurally uncharacterised sequence families

Many of the consortia involved in Phase 1 of the Protein Structure Initiative focused their efforts on the characterisation of prokaryotic targets, often employing a multi-orthologue approach where multiple forms of the same target from related species were fed into the pipeline in order to increase the chances of obtaining expressed soluble protein and ultimately a solved structure [21-23,38-41]. With this in mind we calculated the species distribution within some of the largest structurally uncharacterised Pfam-A and NewFam families, Table 4. We divide the families into four groupings: Eukaryotic, where all family members belong to a eukaryotic genome, viral, where all family members are viral in their origin and prokaryotic. Prokaryotic families are further subdivided into two subgroups; families containing five or more prokaryotic species (for which a multi-orthologue approach could be used) and those containing one or more prokaryotic sequences.

**Table 4:** Analysis of the kingdom distribution.

	**Kingdom distribution of structurally uncharacterised families**		
	**2500 largest Pfam-A**	**All Pfam-A**	**All Pfam-A & Newfam**

**Eukaryotic**	788	1286	8240
**Viral**	295	503	1145
**Prokaryotic (5 or more)**	1381	1959	6290
**Prokaryotic (1 or more)**	1377	2114	8304
**No prokaryotic members**	1125	1833	9488

Analysis of the kingdom distribution of: Column 2, the largest 2500 structurally uncharacterised Pfam-A families Column 3, All structurally uncharacterised Pfam-A families Column 4, All structurally uncharacterised Pfam-A and Newfam families Of the remaining structurally uncharacterised Pfam-A families, 1959 have 5 or more prokaryotic sequences – additional Newfam families can be used to build a larger target list (eg 2500 families shown in Figure 6, black bars).

It is hoped that during PSI2, consortia members will solve in the region of approximately 3000 new structures, much of the effort going towards solving structurally uncharacterised familes. As such, if we consider the largest 2500 non membrane protein domain families with no solved structure (column 2, Table 4), we find that 53% have 5 or more prokaryotic sequences. 31.5% and 11.8% of these families are viral and eukaryotic families respectively, and therefore of minor interest (viral families) or present considerable experimental challenges to most structural genomics groups (eukaryotic families). Solely eukaryotic families present a problem given that the majority of high-throughput structural genomics pipelines are not geared towards eukaryotic proteins, though some groups have put much effort into solving human proteins [42,43].

**Figure 4:**
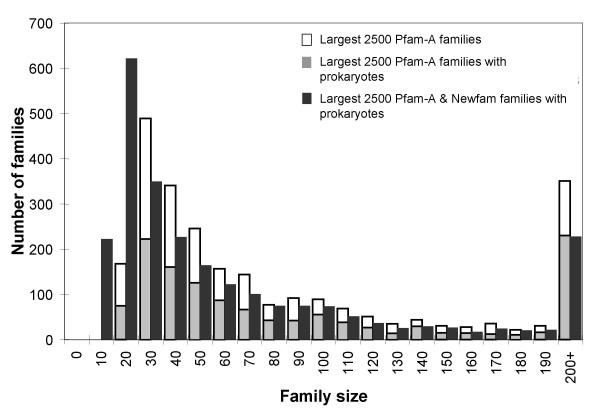
Size distribution of the largest 2500 structurally uncharacterised Pfam-A and NewFam domain families (clear bars) with the proportion of these largest families lacking a prokaryotic sequence (light grey infill on white bars) The size distribution of the 2500 largest Pfam-A and NewFam families with at least five prokaryotic sequences is also shown (black bars).

In Figure 4 we show the size distribution of these 2500 largest, structurally uncharacterised Pfam families (clear bars), with the proportion of these families that are accessible through prokaryotic organisms highlighted (light grey). For comparative purposes we also show the size distribution of the 2500 largest structurally uncharacterised domain families (Pfam-A and NewFam) chosen such that each must contain at least five prokaryotic members (black bars). As shown in Table 4 (column 3) there are 1959 structurally uncharacterised Pfam-A families with five or more prokaryotic sequences. Extra families containing prokaryotic sequences can be selected from the large number of additional NewFam families delineated by the Gene3D resource (Table 4, column 4). The comparative distributions in Figure 4 show that targeting sequence representatives for these more accessible prokaryotic families (black bars) results in the coverage of progressively smaller areas of sequence space – guiding target selection towards comparatively smaller prokaryotic families. Nevertheless, such efforts would still be of significant value, especially if one considers that targeting 2500 of these families would provide structural representatives for 40% of the remaining families in reach of high-throughput structural genomics utilising a multi-orthologue approach.

### Coarse-grained vs fine-grained structural coverage

So far we have described structural coverage according to a coarse-grained measure. That is, we consider a domain sequence to be structurally annotated if it can be assigned to a CATH or Pfam-A_struc family through the use of hidden Markov model searches. In many cases domain sequences will only be very distantly related to the member structure and it is likely that only fold-level inference of structure data can be reliably achieved. Defining the level of detail or granularity of the structural coverage is clearly an important issue for target selection. The domain families identified through CATH or Pfam represent a coarse grained division of sequence space into broad superfamilies containing all relatives sharing a common ancestor. The size of structural families increases dramatically when lowering the threshold for detection structural similarities (i.e. traditional pairwise sequence comparison vs. profile based sequence comparison). Lower thresholds imply lower accuracy of comparative modelling. Therefore the estimate for the number of targets for structural genomics is sensitive to the accuracy we require in comparative modelling to remove a protein from the potential target list.

**Figure 5:**
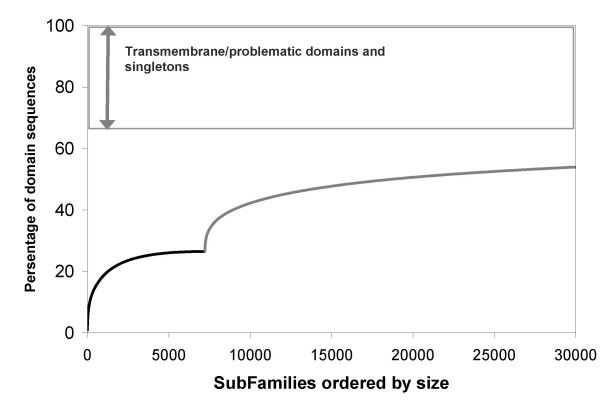
Fine-grained structural coverage of domain sequences in Swiss-Prot-TrEMBL Subfamilies are ranked in order of size, largest to smallest.

In our previous analysis and others [32] it has been shown that in order to achieve reasonable modelling coverage of genome sequences, many orders of magnitude more structures will be required compared to the numbers calculated for coarse-grained structural coverage. In this analysis we applied a greedy-clustering algorithm to group sequences in diverse domain families into closer related (30% sequence identity) subfamilies (see Methods for more detail). Figure 5 shows the structural coverage of those fine-grained subfamilies with a known structure, followed by subfamilies lacking a structure, in descending order of domain family size (i.e. the number of domain sequence members identified through HMM searches). These figures suggest that over 30,000 structures will be required to model half the domain sequences in SP-TrEMBL using a 30% sequence identity modelling cutoff – a huge effort by any standards. However, it is likely that significant advances in threading techniques and comparative modelling will make this a significant over-estimate in terms of structures required.

**Figure 6:**
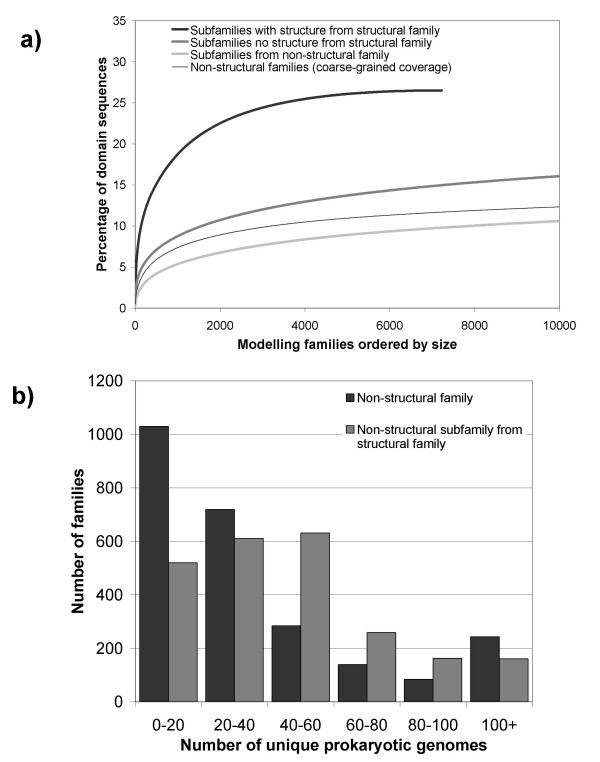
**a) **Comparison of fine-grained structural coverage of subfamilies; subfamilies with a solved structure (black line), non-structural subfamilies within structural families (dark grey line), non-structural subfamilies within non-structural families (light grey line) Also shown in the comparative *coarse-grained *coverage of the largest non-structural families (thin grey line) **b) **Number of unique prokaryotic organisms represented in structurally uncharacterised families (Pfam-A) compared to the number found within structurally uncharacterised subfamilies within CATH and Pfam-A_struc families.

How does solving structures for subfamilies in structurally characterised families compare to solving coarse-grained structurally uncharacterised target families in terms of structural annotation coverage and the relative species distribution of the families? In Figure 6a we show the percentage of domain sequences currently covered by subfamilies that contain a solved structure (black line). This coverage distribution can be compared to the fine-grained sequence coverage that would be gained by solving structures for the largest non-structural subfamilies found in CATH sequence families (dark grey line), and non-structural subfamilies found in structurally *uncharacterised *Pfam families (light grey line). In terms of fine-grained coverage, slightly higher levels of modelling coverage could be achieved by solving the largest fine-grained subfamilies in structural families, such as CATH. However, of greater interest is where we also show the *coarse-grained *coverage of non-structural families in the same figure (thin black line). It is apparent that structural coverage achieved by fine-grained re-sampling in some of the largest subfamilies in CATH families would bring similar levels of additional sequence coverage as targeting structurally uncharacterised coarse-grained Pfam families.

In addition we also compare the number of unique prokaryotic genomes found within structurally uncharacterised coarse-grained *families *and non-structural fine-grained *subfamilies *within structural families, Figure 6b. Interestingly, the structurally uncharacterised subfamilies in CATH families tend to have a wider distribution across the prokaryotic genomes and might therefore be considered as more attractive targets from the viewpoint of an experimentalist.

Solving a representative structure for as yet structurally uncharacterised domain families forms a significant cornerstone of PSI structural genomics initiatives. Such families, if targeted carefully, are likely to represent proteins with previously unobserved folds and/or functions. However, it is also apparent that there exists a diminishing law of returns if one is to measure the contribution of structural genomics on the additional contribution to coarse-grained structural coverage. We have seen that a significant proportion of domain sequences belong to a relatively few large domain families. Beyond these families there exist a considerable number of smaller families from which target lists can be derived. How we access these families efficiently requires considerable thought if one considers their accessibility through prokaryotic organisms, especially so for experiments utilising a multi-orthologue approach. Some structurally uncharacterised Pfam families may be very diverse relatives of known structural families in CATH and are therefore of interest because they may reveal the extent to which families can diverge, illuminating the structural plasticity of these families.

**Figure 7:**
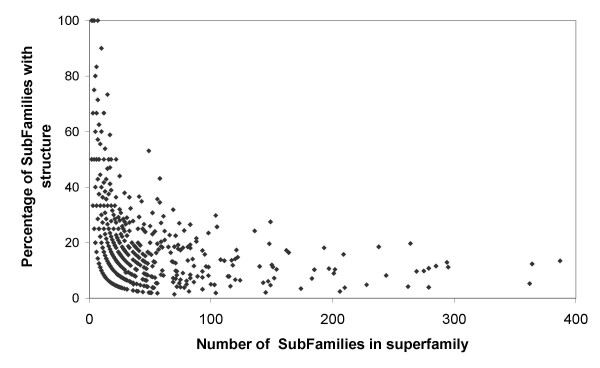
The number of subfamilies in structural families against the number of those subfamilies with a solved structure Our structural and functional understanding of many of the most diverse domain families would benefit from the structural characterisation of additional family members.

Structural genomics is complementary to traditional biology as there is a greater interest in solving the structures of proteins whose function is not yet known. It is hoped that once solved, a given structure will lend itself to computational function prediction methods, where function can be inferred or predicted. The largest domain families contain a considerable proportion of genome sequences but they also contain a considerable proportion of the sequence diversity of genome sequences. As can be seen on Figure 7, much of this sequence diversity lacks any close structural homologue. A website detailing the structural coverage of CATH superfamily domain sequence relatives has been made available [53]. Some protein families are quite well conserved in function during evolution. Clearly, fewer targets will be needed from these families. However other families are extremely diverse, for example relatives in the P-loop hydrolase superfamily exhibit more than 40 functions [34].

## Conclusion

Recent analyses [3,4] have shown that structural genomics projects are already making significant contributions to our knowledge of structure space in terms of number and value of structure depositions. Despite this, structural genomics will clearly not be able to support the experimental determination of structures of all proteins. Accordingly target selection should therefore sample broadly from family space in a manner that optimises the number of genome sequences that can be modelled. The proposal of a Pfam5000-like strategy gives an effective approach to the selection of target families for which a structure has not yet been solved. Whilst conceptually simple, certain considerations must be addressed when putting the system into practice. As has been shown, many of the largest structurally uncharacterised Pfam families have few or no prokaryotic members, whilst the size distribution of these families tends towards smaller families (in comparison to the structurally characterised families). Nonetheless, the characterisation of such families should play a significant part in structural genomics, especially in light of the identification of novel folds or new superfamilies. In addition, it may also be valuable to choose additional targets from large structurally characterised families which so far have a very low level of fine-grained homology modelling coverage.

Comparative genome analysis has shown that many of the most functionally diverse domain superfamilies have expanded significantly during evolution through extensive gene duplication within a genome [54,55]. Following domain duplication evolution of a new function has been achieved in a number of ways including fusion of the duplicate domains with a range of different domain partners. Other mechanisms include the significant structural embellishment of a domain or changes in the oligomerisation state of a protein [44]. Increasing the coverage of structure annotations will reveal new insights between protein sequence, structure and function, which in turn will expedite our understanding of protein function on the molecular level and improve the methods by which we can automatically provide structure-guided functional annotations to new protein structures [56].

## Methods

### Assignment of CATH, Pfam-A and NewFam domain family annotations

A non-redundant set of protein sequences were taken from the Swiss-Prot version 48.1 and TrEMBL version 31.1 databases [45] with sequences shorter than 50 residues excluded, giving a dataset of 2,241,277 sequences. Domain families were taken from the Gene3D database which includes domain annotations from the CATH [25] and Pfam [27] domain classifications as well as Gene3D NewFam families [32]. CATH version 3.0 domain assignments (representing 2043 CATH domain superfamilies) were made by searching libraries of hidden Markov models (HMMs) against the sequence dataset using HMMer [48] where an HMM match was assigned using an E-value cutoff of 0.01. Overlapping annotations were resolved using the DomainFinder algorithm [57]. Pfam assignments were based on Pfam version 19 which classifies 8193 Pfam-A families. The HMMer protocol was used to identify family members using the family-specific gathering threshold cutoff to identify true-matches.

To gain a maximum coverage of protein families, regions without CATH or Pfam-A assignments were further annotated, where possible, by NewFam families. As described previously [32] Gene3D NewFam families are automatically generated from the TribeMCL [58] clustering of whole or partial sequences to which no CATH or Pfam-A domain assignment can be made. Such unassigned regions have been shown to follow a domain-like length distribution [32], Figure S1 Supplementary Material, where the largest NewFam cluster contains 453 sequences.

### Resolving overlapping CATH and Pfam-A families

A hierarchical approach to domain assignment was applied where CATH and Pfam domain annotations are found to overlap. A hybrid of CATH and Pfam assignments were calculated where CATH domain matches were given priority over Pfam domain matches. Domains in the CATH database are identified from both sequence and structure, which is generally considered to be a more reliable approach for protein domain delineation than their identification from sequence. Conflicts were resolved according to the degree of sequence overlap and family overlap: In cases where 70% or more of the sequences in a Pfam family were found to overlap a CATH family by 70% or more of their sequence length, the Pfam family was inherited into the CATH family. In cases were less than 70% of the Pfam sequences members overlapped the CATH family, the non-overlapping remainder of the Pfam family was assigned as a structurally uncharacterised Pfam family remainder. In some cases, where a partial region of a Pfam family was overlapped by a CATH assignment, a domain-like (in terms of length) Pfam region remained. A cutoff of 80 residues was used to filter such remaining Pfam regions, which were subsequently assigned to Pfam sub-domain families. On average, 1.8 Pfam families were merged into each CATH domain family. In all cases family size was defined as the number of unique sequences matching each domain family.

### Families matching solved structures

Since the CATH database is partially reliant on manual curation, it is not entirely up to date with the PDB. In order to address this fact, we searched sequences from Pfam-A (after overlap with CATH families had been resolved) against all proteins deposited into the PDB up to the 21^st ^January 2006. Pfam families matching a PDB structure were defined as Pfam_struc.

### Completed genomes

Analysis on completed genomes was based on genome-sequence sets defined by Integr8 [50]. We used 263 completed genomes (913,094 sequences) in this study, 237 prokaryotes and 26 eukaryotes, each of which had 95% or more of their sequences included in the Swiss-Prot&TrEMBL database. We refer to this completed genome sequence dataset as integr8-263.

### Species data

Taxonomic sequence data for each protein in Swiss-Prot and TrEMBL was taken from the UniProt Knowledge database version 8.0 [45].

### Identification of helical transmembrane and problematic sequences and families

The Memsat program [59] was used to identify transmembrane helices using default thresholds. We used the COILS2 algorithm [60] to identify coiled-coil regions, using a probability cutoff of 0.9 and a window size of 28 residues and the SEG program [61] with default parameters to identify regions of low complexity. Helical transmembrane families were defined as those with 30% or more members having one or more helical membrane regions predicted by the Memsat algorithm. Problematic families were defined as those in which 30% or more members had one or more low-complexity regions (15 or more residues in length) as annotated by SEG or with a coiled-coil prediction.

### Greedy coverage algorithm to identify fine-grained sequence clusters

A greedy coverage algorithm was run on sequence relatives assigned to CATH and Pfam domain families as follows: Links between family members were assigned, using an implementation of the Needleman and Wunsch global alignment sequence comparison algorithm, in cases where sequence identity and overlap were found to be = 30% and = 80% respectively. The sequence (representing a possible homology modelling template) with the highest number of links is first selected, and removed, along with all those sequences to which it is linked, from further calculations, to form a new cluster. This step is repeated until no sequences are left in the family. In cases where a family contained one or more experimentally solved structure a slightly different procedure was employed. First, each structurally characterised sequence was used to seed each cluster, identifying true families of sequences that can be homology modelled, after which the general method was implemented.

### Identifying structural relatives for Pfam families

In order to calculate the number of newly structurally characterised Pfam families that represented a new fold or superfamily we identified the oldest PDB structure (in terms of release date) matching each Pfam family. Pfam-A HMMs were searched using the HMMer protocol against two sequence datasets representing the CATH domain database (version 3.0) and the SCOP domain database (version 1.69). In mind of the fact that each database falls behind the PDB care was taken to identify whole sequences or domain sequences (from partially classified chains) not yet assigned into the respective classifications. Classified domains and unclassified sequences were combined to produce a representative sequence set for each database containing all PDB chains released up to 31^st ^December 2005. COMBS sequences [58] were used for the CATH domain database and ASTRAL sequences for the SCOP database (24). Seqres sequences were used for unclassified PDB sequences (49). The HMMer protocol was used to search the Pfam HMM library using Pfam specific gathering thresholds.

## Authors' contributions

RLM carried out the study, participated in its design and drafted the manuscript. TEL participated in data acquisition and analysis. CAO helped conceive the study, assisted in its design and in drafting the manuscript. All authors read and approved the final manuscript.
